# Circulating CD16-Positive Monocyte-like Myeloid-Derived Suppressor Cells and Intermediate Monocytes Associated with Clinical and Immunological Complications in Pars Planitis Patients

**DOI:** 10.3390/cells14201636

**Published:** 2025-10-21

**Authors:** Agata Kosmaczewska, Joanna Przeździecka-Dołyk, Lidia Ciszak, Zofia Rojek-Gajda, Irena Frydecka, Anna Turno-Kręcicka, Marta Misiuk-Hojło, Edyta Pawlak

**Affiliations:** 1 Laboratory of Immunopathology, Department of Experimental Therapy, Hirszfeld Institute of Immunology and Experimental Therapy, Polish Academy of Sciences, 53-114 Wroclaw, Poland; 2 CREO Research and Development Centre, SPEKTRUM Clinical Ophthalmic Centre, 53-334 Wroclaw, Poland; joanna.przezdziecka.dolyk@gmail.com; 3 Deanery of Clinical Sciences, College of Medicine and Veterinary Medicine, University of Edinburgh, Edinburgh EH16 4TJ, UK; 4 Dental Center STOMO Clinic, 53-411 Wroclaw, Poland; 5 Department and Clinic of Ophthalmology, Faculty of Postgraduate Medicine, Medical University of Wroclaw, 50-556 Wroclaw, Poland; 6 4th Military Clinical Hospital with Polyclinic SPZOZ, 50-981 Wroclaw, Poland

**Keywords:** pars planitis/intermediate uveitis, cystoid macular edema, disease severity, intermediate monocyte, myeloid-derived suppressor cell

## Abstract

Recently, we observed that pars planitis (PP) patients present alterations in peripheral blood (PB) Th17/Treg associated with dysregulation in the Th1 response. Yet, little is known about the systemic distribution of myeloid cells, which drive the recruitment and differentiation of the adaptive effectors toward pathogenic inflammatory Th1 and Th17 as well as regulatory lymphocytes in PP. Although myeloid populations in patients with uveitis have previously been addressed, the data did not provide an exact description of PP patients. Using flow cytometry, we evaluated monocyte and IDO-expressing monocyte-like myeloid-derived suppressor cell (MDSC) subpopulations in PB samples from 15 patients with different courses of PP (cystoid macular edema and non-macular edema subgroups; CME and nCME, respectively) and 17 healthy controls (HCs) in relation to the Th1, Th17, and immunoregulatory subsets. We observed that only PP patients from the CME subgroup presented a significantly higher fraction of CD16^+^ IDO-expressing MDSCs and intermediate CD14^high^CD16^+^ monocytes compared to the HCs; this corresponded with relative up-regulation of Th1 and Th17, and down-regulation of Treg. In addition, alongside the increased percentage of IDO-expressing CD16+ MDSCs, the MDSC compartment displayed an inappropriate level of IDO (more pronounced in the CD16^−^ subset) only in CME patients. At the same time, the fraction of CD16^−^ myeloid cells did not differ significantly among the patient cohorts and healthy participants. Our study is the first to evaluate subpopulations of circulating myeloid cells in PP patients and indicates that an increased fraction of CD16^+^ myeloid cells might reflect the immunological and clinical severity of PP.

## 1. Introduction

Pars planitis (PP) is an intermediate uveitis of unknown etiology [1]. According to the Standardization of Uveitis Nomenclature (SUN) Working Group, pars planitis represents a subset of intermediate uveitis characterized by the presence of snowbanks and snowball formation (the fibro-inflammatory aggregates) on the pars plana and within the vitreous in the absence of any systemic or infectious disease [1]. So far, the vast majority of research focused on intermediate uveitis, while only a few articles have been devoted to PP as a separate disease entity, suggesting its immunopathogenic background.

In fact, it is a chronic intraocular T cell-mediated inflammatory disorder, in which vitreous, pars plana, and peripheral retina represent major sites of inflammation. In recent years, increasing interest in the role of the innate immune system (including myeloid cells) in the pathogenesis of uveitis has been reported. The murine model of experimental autoimmune uveitis (EAU) is utilized as an experimental model for investigating the underlying mechanisms of uveitis [2]. Previous research based on EAU has shown that during the early phase of ocular inflammation, peripheral monocytes, as systemic precursors of macrophages are innate effectors in the inflammatory responses through secretion of pro-inflammatory cytokines, such as TNF-α, IFN-γ, IL-1β, and IL-6, which lead to injury to the surrounding tissue and initiation of EAU [3,4,5,6,7,8]. Activation of adaptive immunity is a critical step during the progression of inflammation in the amplified phase of uveitis [4,9,10]. In accordance, at this stage of inflammation, the cytokine milieu creates a microenvironment supporting the recruitment and differentiation of CD4+ T lymphocytes toward pathogenic inflammatory Th1 and Th17 cells in the retina. The release of Th1/Th17 cytokines activates inflammatory cascades, which disrupt the blood–retina barrier (BRB) and contribute to local tissue damage. In addition, Th1 and Th17 cells secrete chemokines attracting other inflammatory cells, such as monocytes, granulocytes, and non-specific lymphocytes responsible for further inflammation and destruction of ocular tissues. In the eye, the ocular microenvironment normally favors T cell differentiation to a regulatory phenotype, thereby maintaining ocular immune privilege. Likewise, regulatory CD4^+^ and CD8^+^ T cell (respectively, Treg and Tsup), regulatory B cell (Breg), and myeloid-derived suppressor cell (MDSC) subsets were shown to limit immune activation in the eye. Recent data shows that during PP, inflammatory leukocyte infiltration is accompanied by evidence of BRB breakdown, hence indicating down-regulation in its immunomodulatory capacity [11]. Compartmentalization of autoantigens in the eye disrupts peripheral immune tolerance mechanisms as well, resulting in the exacerbation of uveitis. Immunopathogenic background of PP, including the role of adaptive immune responses, was examined and described in detail in our previous research [12,13].

Evidence from numerous human studies suggests that an increase in PB and tissue-infiltrating monocytes could be an indicator of the severity and/or activity of inflammatory diseases [14,15,16]. In humans, monocytes have been subdivided by the expression of CD14 and CD16 surface markers into three different subtypes named classical (CD14^high^CD16^−^), non-classical (CD14^+^CD16^high^), and intermediate (CD14^high^CD16^+^) [17]. Given the expansion of intermediate CD14^high^CD16^+^ monocytes in many inflammatory and autoimmune disorders, this monocyte subset remains of important clinical interest. Cluster analysis has revealed that the intermediate monocyte subset is closely related to CD16^−^ classical monocytes and displays pro-inflammatory properties [18], such as secretion of high amounts of TNF-α in response to LPS stimulation [19]. In turn, monocytic-like myeloid-derived suppressor cells (MDSCs) is a population with the capacity to suppress the T cell responses involved in tumors as well as non-infectious uveitis and other inflammatory disorders [20,21,22,23]. It is well documented that MDSCs can promote immunosuppression via the indoleamine 2,3-dioxygenase (IDO)-pathway [24,25,26,27]. IDO expression by MDSCs displays an inhibitory effect by the combined reduction in tryptophan and induction of toxic metabolites known as kynurenine, which are critical for Treg generation and impaired Th1/Th17 responses [28,29].

In our recent work, we demonstrated that the imbalance in PB Th17/Treg in PP patients is associated with dysregulation of IFN-γ-secreting Th1 cells and that these alterations in adaptive immune responses could be attributed to patients with clinical complications [12]. Taking into account that the development and recurrence of PP depends on a complex interplay between the elements of innate and adaptive immunity, and that the role of myeloid cells such as monocytes and MDSCs has not been evaluated in PP so far, our present goal was to investigate the distribution of circulating myeloid cells in PP patients in relation to the immunological parameters of adaptive immunity (Th1, Th17, Treg) and the clinical course of the disease (with or without CME). The pathogenesis of CME, which is a major cause of visual loss in PP, includes retinal vascular hyperpermeability and endothelial cell damage leading to vascular leakage. CME can be caused by a variety of factors released by immune cells involved in the innate immune response [13,30]. We herein observed for the first time that circulating CD16^+^ monocytes and IDO-expressing CD16^+^ MDSCs were increased in PP patients, primarily those with CME. At the same time, the fraction of CD16^−^ myeloid cells did not differ significantly among patients and healthy participants. Our study indicates the involvement of myeloid cells in the pathogenesis and severity of PP.

## 2. Materials and Methods

### 2.1. Participants

In our analyses, the study group comprised 15 patients with PP. All the patients enrolled for screening were evaluated by way of a full ophthalmological examination, a summary of which (including the grading scales and specifications of the equipment used) is provided in the Supplementary Materials (Table S1). A clinical summary of additional tests is presented in Table S2. In accordance with the Standardization of Uveitis Nomenclature (SUN) Working Group criteria for the diagnosis of PP, in all included patients, both the systemic and infectious causes of the uveal manifestation were excluded [1]. The full inclusion and exclusion criteria for patients are presented in Table S3. According to the screening and recruitment protocol, 14 out of 29 patients were excluded from the final analysis. Considering the clinical course, the patients were finally subdivided into macular edema (n = 9) and non-macular edema (n = 6) subgroups (CME and nCME, respectively).

The control population comprised 17 healthy individuals matched for age and sex, with no medical history affecting the immune system such as diabetes, autoimmune diseases (including connective tissue diseases), malignancies, or chronic or acute infections, and without any treatment affecting the immune system for six months before beginning the study. The project protocol was referred to the local Bioethics Committee and approved (number: KB-329/2014). The trial was performed in compliance with the provisions of the Declaration of Helsinki, as well as the International Conference on Harmonization Good Clinical Practice guidelines and local regulations. Before any medical procedure was performed, every participant signed two copies of the written informed consent and was given one of them.

### 2.2. Cytometric Analysis

Peripheral blood mononuclear cells (PBMCs) were isolated by buoyant density–gradient centrifugation on a Lymphoflot (Biotest, Dreieich, Germany) from 40 mL of freshly drawn peripheral venous blood. PB mononuclear cells (PBMCs) were stained with several combinations of anti-human fluorochrome-conjugated monoclonal antibodies (mAbs) for three-color analysis.

The percentages of cytokine-producing T cells (Th1 and Th17) were calculated after stimulation with 25 ng/mL phorbol 12-myristate 23-acetate (PMA, Sigma-Aldrich, Darmstadt, Germany) and 1 µg/mL of ionomycin (Ion) (Sigma-Aldrich, Darmstadt, Germany) in the presence of 10 µg/mL of brefeldin A (BFA, a protein transport inhibitor) (Sigma-Aldrich, Darmstadt, Germany) for 4 h at 37 °C in a humidified atmosphere containing 5% CO_2_. Because incubation with PMA triggers internalization and degradation of the CD4 molecule, which would affect the identification of Th1 (phenotyped as CD4^+^IFN-γ^+^) and Th17 cells (characterized as CD4^+^IL-17^+^) [31], both subpopulations were identified as CD3+CD8^−^IFN-γ^+^ and CCR4^−^CXCR3^+^IFN-γ^+^ as well as CD3^+^CD8^−^IL-17^+^ and CCR4^+^CCR6^+^IL-17^+^ cells, respectively. Directly after PMA+Ion stimulation, the PBMCs were surface-stained with the respective mAbs as follows: anti-CD3/PerCP (BD Biosciences, San Diego, CA, USA), anti-CD8/PE (BD Biosciences, San Diego, CA, USA), anti-CCR4/PerCP (BioLegend, San Diego, CA, USA), anti-CXCR3/PE (BioLegend, San Diego, CA, USA), and anti-CCR6/PE (BioLegend, San Diego, CA, USA). Then, after fixation and permeabilization with the Fixation/Permeabilization Buffer Set (eBioscience, San Diego, CA, USA), the cells were incubated with anti-IFN-γ/FITC (BD Biosciences, San Diego, CA, USA) or anti-IL-17/FITC (BioLegend, San Diego, CA, USA) mAbs for 30 min in the dark at room temperature.

For analysis of the immunoregulatory lymphocyte subpopulations, including Treg (phenotyped as CD4^+^CD25^+^CD127^−^ and CD4^+^CD25^high^CD127^−^ cells), Tsup (identified as CD8^+^CD28^−^FOXP3^+^ cells), and Breg (characterized by the CD19^+^CD24^high^CD38^high^ phenotype), PBMCs were aliquoted into tubes directly after isolation for further staining with the following mAbs: anti-CD4/PerCP (BD Biosciences, San Diego, CA, USA), anti-CD25/FITC (BD Biosciences, San Diego, CA, USA), anti-CD127/PE (BioLegend, San Diego, CA, USA), anti-CD19/FITC (BD Biosciences, San Diego, CA, USA), anti-CD24/PerCP (BioLegend, San Diego, CA, USA), anti-CD38/PE (BD Biosciences, San Diego, CA, USA), anti-CD8/PerCP (BD Biosciences, San Diego, CA, USA), and anti-CD28/FITC (BD Biosciences, San Diego, CA, USA), respectively. For intracellular staining, the cells were then fixed and permeabilized with the Fixation/Permeabilization Buffer Set (eBioscience, San Diego, CA, USA) according to the manufacturer’s instructions, with subsequent incubation with anti-human FOXP3/PE (BD Biosciences, San Diego, CA, USA) mAbs for 30 min in the dark at room temperature.

For analysis of the myeloid cell populations (both monocytes and monocytic-like MDSCs), the PBMCs were aliquoted into tubes directly after isolation for further staining with anti-CD14/FITC (BioLegend, San Diego, CA, USA) and anti-CD16/PerCP (BioLegend, San Diego, CA, USA) mAbs and incubated for 30 min in the dark at room temperature. Monocyte subpopulations (classical CD14^high^CD16^−^, intermediate CD14^high^CD16^+^, and non-classical CD14^+^CD16^high^) were calculated relative to the fluorescence intensity of CD14 and CD16 surface markers. For analysis of IDO-expressing MDSC subpopulations (M-MDSC phenotyped as CD14^+^CD16^+^ and CD14^+^CD16^−^ subsets [20]), the PBMCs were surface-stained with anti-CD14/FITC (BioLegend, San Diego, CA, USA) and anti-CD16/PerCP (BioLegend, San Diego, CA, USA) mAbs and incubated for 30 min in the dark at room temperature. Then, for intracellular staining, the cells were fixed and permeabilized with the Fixation/Permeabilisation Buffer Set (eBioscience, San Diego, CA, USA) according to the manufacturer’s instructions, followed by incubation with anti-human IDO/PE (eBioscience/ImmunoGen, San Diego, CA, USA) mAbs for 30 min in the dark at room temperature.

Directly after immunostaining, the cells were washed and analyzed by flow cytometry using a FACScan cytometer (Becton Dickinson, BD Biosciences, San Diego, CA, USA) equipped with Cell Quest software 2.0 (BD Biosciences, San Diego, CA, USA). Appropriate fluorochrome-labeled isotypic controls were used for gate settings in each case. A total of 30,000 events were recorded for each sample before any electronic gate setting. The gating strategy is presented in Figure S1 in Supplementary Materials.

### 2.3. Statistical Analysis

Statistical analysis was performed using the Statistica 7.1 package and GraphPad Prism 8.01. For all analyzed variables, the median values and 25th and 75th interquartile ranges (IQ ranges) were calculated. All collected data were examined for normal distribution and homogeneity of variances using the Shapiro–Wilk test and Levene’s test, respectively. If data were normally distributed and had homogeneous variances, Student’s t-test or one-factor analysis of variance (ANOVA) followed by a post hoc test (Scheffe’s test) was used to identify a statistically significant difference for the primary hypothesis of differences in cell subpopulations between the study groups. If data were not normally distributed and/or had heterogeneous variances, the Mann–Whitney U test or Kruskal–Wallis one-way analysis of variance by ranks followed by a post hoc test (Dunn’s test) was applied. The relationship between the analyzed variables was tested with the Spearman’s or Pearson’s rank correlation coefficient. In all analyses, differences were considered significant when *p* ≤ 0.05.

## 3. Results

### 3.1. Adaptive Immune Cells (Pro- and Anti-Inflammatory Lymphocytes)

As we recently observed that active PP is characterized by altered PB pro-inflammatory and anti-inflammatory T cells, including Th1, Th17, and Treg [12], we decided to extend our research by analyzing two subgroups of PP patients classified as those with or without cystoid macular edema (CME or nCME, respectively), reflecting the severity of ocular inflammation. Regarding the clinical course, we found substantially lower proportions of the Th1 subsets (defined as CD3^+^CD8^−^IFN-γ^+^ and CCR4^−^CXCR3^+^IFN-γ^+^ cells) in both cohorts of patients compared to the HCs (*p* ≤ 0.05; Table 1). Although there were no significant differences in Th1 cell frequency between the CME and nCME patient groups, some detectable differences were seen; alongside a deficit in Th1 population in all patients, those with CME exhibited a slightly higher level of PB Th1 cells than nCME patients.

**Figure 1:**
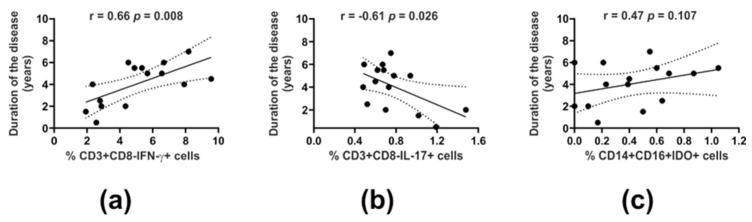
Associations of circulating innate and adaptive cell effectors with the clinical course of PP. Correlations of disease duration with the proportions of PB CD3^+^CD8^−^IFN-γ^+^ (Th1) (**a**), CD3^+^CD8^−^IL-17^+^ (Th17) (**b**), and CD14^+^CD16^+^IDO^+^ cells (IDO-expressing CD16^+^ MDSCs) (**c**).

Moreover, Th1 frequency was also found to correlate with the duration of the disease (r = 0.66, *p* = 0.008) (Figure 1a). Considering the Th17 subset (identified as CD3^+^CD8^−^IL-17^+^ and CCR4^+^CCR6^+^IL-17^+^ cells), we noted a substantially higher population of PB IL-17^−^ secreting cells in PP patients in comparison with the corresponding cells in the HCs (*p* < 0.001) (Table 1), and this was observed irrespective of disease severity (CME vs. nCME, *p* = 0.999). Unlike Th1, we observed a negative association of the Th17 subset with disease duration (r = −0.61, *p* = 0.026) (Figure 1b).

Simultaneously, in patients with CME or nCME, and the HCs, we evaluated the size of the different regulatory lymphocyte subpopulations, including CD4^+^ T regulatory cells (Treg, phenotyped as CD4^+^CD25^+^CD127^−^ and CD4^+^CD25^high^CD127^−^), CD8^+^ T suppressor cells (Tsup, identified as CD8^+^CD28^−^FOXP3^+^), and B regulatory cells (Breg, described as CD19^+^CD24^high^CD38^high^). Compared to the HCs, we recently found a loss of the median proportion of the patients’ CD4^+^ and CD8^+^ immunoregulating T cells [12].

Current analysis based on the clinical course revealed that, among patients, Treg and Tsup deficiency was limited mainly to the CME group when compared to HCs (*p* ≤ 0.045), but it was not seen in nCME patients (Table 1). Considering the Breg compartment, the differences among patient cohorts and HCs were not statistically significant (Table 1). When the duration of the disease was analyzed, no correlation was found with any examined subpopulation of Treg, Tsup, or Breg lymphocytes.

### 3.2. IDO-Expressing MDSCs

Although MDSCs were shown to play an essential role in the regulation of immune responses by suppressing activated T cells and expanding regulatory T cells via IDO expression [32,33], their role in mediating the resolution of inflammation is still conflicting [22,34,35]. In humans, MDSCs can be isolated from monocytes and neutrophils based on phenotypic markers; however, there is still no unified standard for surface indices [21]. Since monocytic MDSC (M-MDSC), identified in humans as CD14^+^CD16^+^ and CD14^+^CD16^−^ cells [20], were found to be enriched in autoimmune non-infectious inflammatory conditions [36,37], the aim was to assess the frequency of IDO-expressing CD16^+^ and CD16- MDSCs in the PB of PP patients in relation to different severities of ocular inflammation in comparison to the values obtained from the HCs.

**Figure 2:**
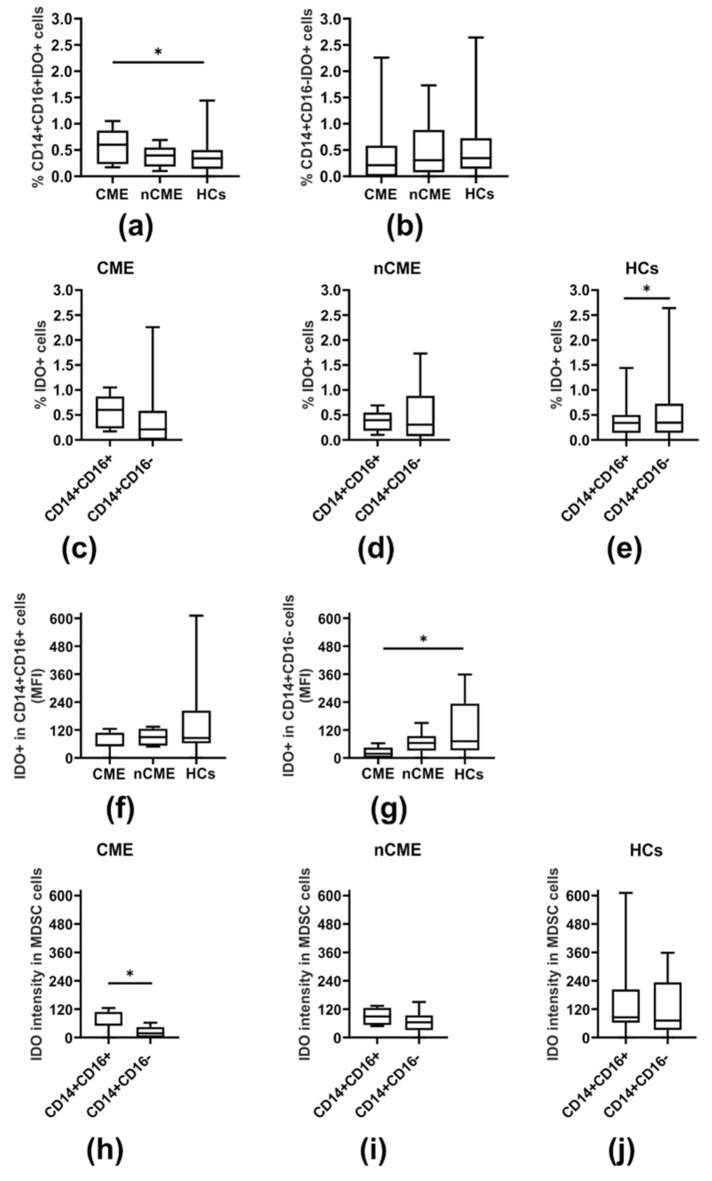
IDO expression in PP patients and healthy donors. (**a**,**b**) Box plots show the proportions of IDO-expressing CD14+CD16+ MDSCs (**a**) and IDO-expressing CD14+CD16-MDSCs (**b**) in PP patients with CME, without CME (nCME), and HCs. (**c**–**e**) Comparison of IDO expression between CD16+ and CD16- MDSC subsets in PP patients with CME (**c**), without CME (nCME) (**d**), and HCs (**e**). (**f**,**g**) The quantitative analysis of IDO expression presents the mean fluorescence intensity (MFI) of IDO in CD14+CD16+ MDSCs (**f**) and CD14+CD16- MDSCs (**g**). (**h**–**j**) Box plots show the level of IDO (MFI) in CD14+CD16+ and CD14+CD16- MDSC subsets in patients with CME (**h**), without CME (nCME) (**i**), and HCs (**j**). Boxes and whiskers show the 25th and 75th interquartile range and minimum–maximum, respectively; the median is the central line in each box. * indicates 0.001 < *p* ≤ 0.05.

Compared to HCs, we observed a significantly increased fraction of CD16^+^IDO^+^ MDSCs in the PP cohort (*p* = 0.011) (Table 2). This was in contrast to CD16^−^IDO^+^ MDSCs, which reached comparable levels in all individuals studied (Table 2). Regarding the clinical course, we noted that the increase in CD16^+^IDO^+^ MDSCs was assigned to the CME subgroup (*p* = 0.037) (Figure 2a, Table 2), whereas nCME patients displayed a statistically similar frequency of CD16^+^IDO^+^ MDSCs compared with corresponding cells from CME patients or HCs (Figure 2a, Table 2). In turn, the proportions of CD16^−^IDO^+^ MDSCs did not exhibit statistically significant differences among the studied groups of patients and HCs (Figure 2b, Table 2). When comparing CD16^+^IDO^+^ and CD16^−^IDO^+^ MDSC subsets in all participants studied (Figure 2), we noted an increasing expression of IDO within CD16^+^ MDSCs in patients with CME (*p* = 0.028) (Figure 2h), which was in contrast to a predominance of an IDO-expressing CD16^−^ MDSC fraction in HCs (*p* = 0.016) (Figure 2e); there was no difference found in the nCME group (Figure 2d,i).

**Figure 3:**
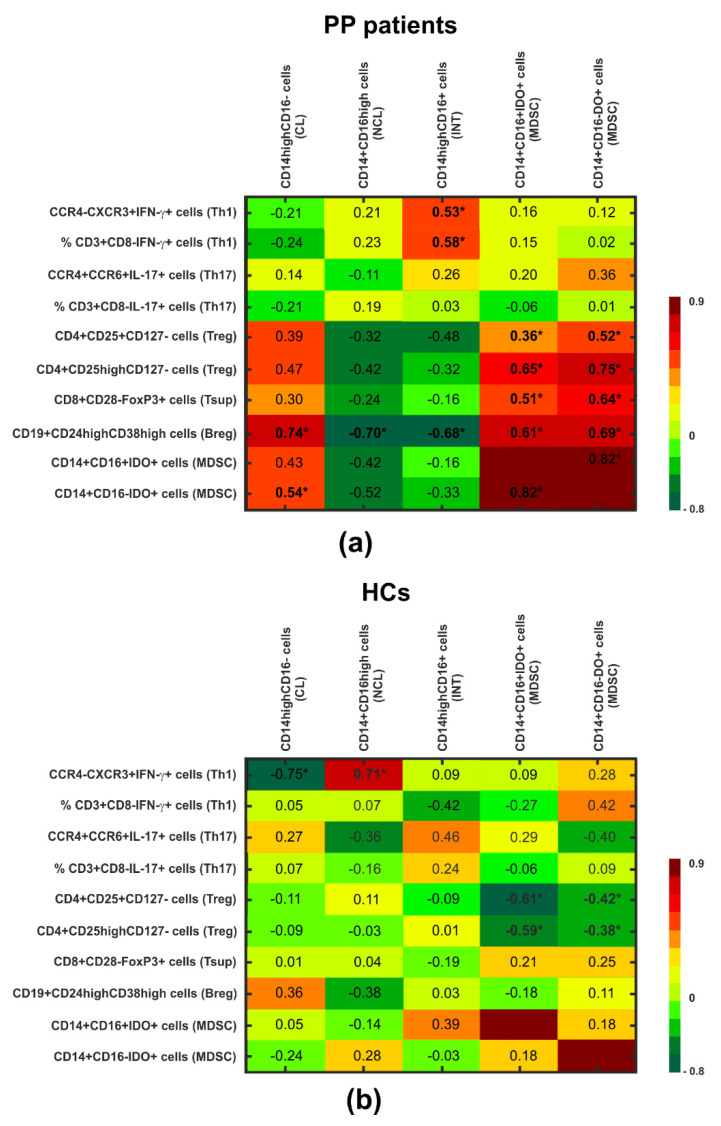
Heat map of the Spearman’s rank order correlations between the studied cell populations in PP patients (**a**) and HC group (**b**). Negative correlation was pointed in green and positive correlation was pointed in red. The darker the color, the stronger the correlation intensity. The numbers represent the correlation coefficient. * indicates 0.001 < *p* ≤ 0.05.

Notably, quantitative analysis of IDO expression for the whole patient cohort revealed a tendency toward lower levels in patient MDSCs, more pronounced in the CD16- fraction, in comparison to the corresponding cells in HCs (*p* = 0.071) (Table 2). Further analysis considering the clinical severity of PP led to indications that deficient levels of IDO in MDSCs were observed mainly in CME patients in both the CD16+ and CD16- subpopulations; however, the difference was of statistical significance only with regard to the amount of IDO in the CD16^−^ MDSC subset compared to HCs (*p* = 0.041) (Figure 2g, Table 2). Also, patient IDO-expressing MDSCs (both CD16+ and CD16-) were found to positively associate with all the immunoregulatory T and B cells studied (*p* < 0.05) (Figure 3a), which was in stark contrast to the HCs, for whom negative correlations of IDO^+^ MDSCs were noted for only the Treg subsets (*p* < 0.05) (Figure 3b). Also, we did not find any significant correlation between the duration of PP and IDO^+^ MDSCs. However, a slight tendency of CD16^+^IDO^+^ MDSCs to associate with disease duration was noted (r = 0.47, *p* = 0.107) (Figure 1c).

### 3.3. Monocyte Subpopulations

**Figure 4:**
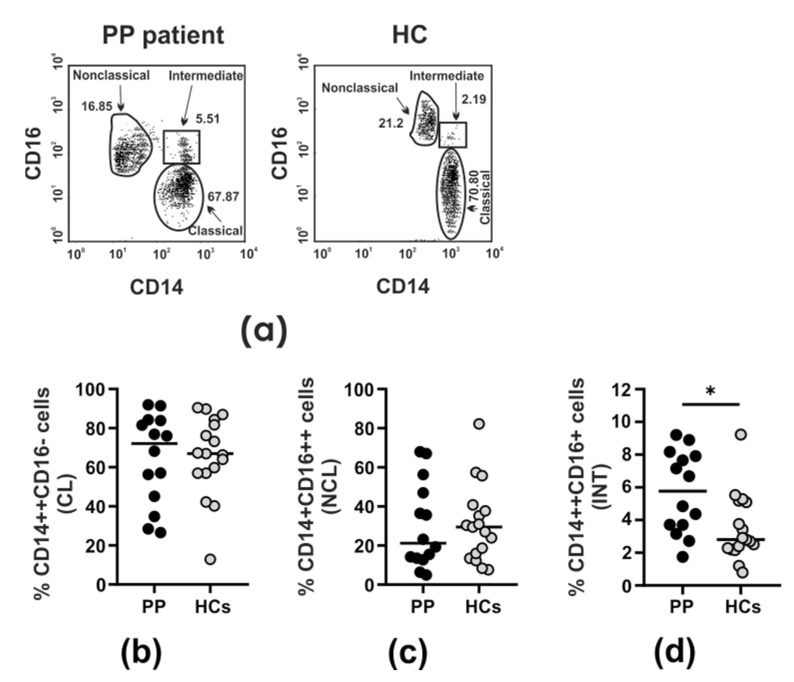
Monocyte subpopulations in the PB of PP patients and healthy donors. PB mononuclear cells were gated using FSC/SSC profiles followed by gating on CD14^+^ cells to identify the monocyte subpopulations. Dot plots show the proportions of classical CD14^high^CD16^−^ monocytes, non-classical CD14^+^CD16^high^ monocytes, and intermediate CD14^high^CD16^+^ monocytes in representative PP patients and HCs (**a**). Box plots show the proportions of classical CD14^high^CD16^−^ monocytes (CL) (**b**), non-classical CD14^+^CD16^high^ monocytes (NCL) (**c**), and intermediate CD14^high^CD16^+^ monocytes (INT) (**d**) in the whole group of PP patients irrespective of the clinical course compared to HCs. The horizontal lines show the median values. * indicates 0.001 < *p* ≤ 0.05.

As skewed monocyte subsets have been reported in autoimmune inflammatory disorders [38,39,40,41,42,43], we aimed to find out whether this relationship also exists in the context of non-infectious intermediate uveitis. Three subsets of monocytes, including classical CD14^high^CD16^−^, non-classical CD14^+^CD16^high^, and intermediate CD14^high^CD16^+^, were defined according to the expression level of CD14 and CD16. Representative dot plots showing the CD14 and CD16 expression on monocytes in the particular monocyte subsets in PP patients and HCs are shown in Figure 4a.

**Figure 5:**
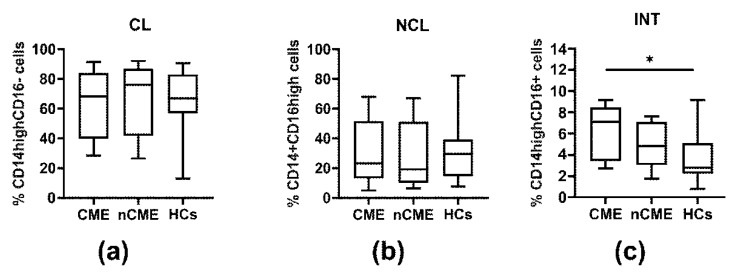
Distribution of monocyte subpopulations in the cohorts of PP patients and HCs. Box plots show the peripheral frequencies of classical CD14^high^CD16- monocytes (**a**), non-classical CD14^+^CD16^high^ monocytes (**b**), and intermediate CD14^high^CD16^+^ monocytes (**c**) in patients with CME, without CME (nCME), and HCs. Boxes and whiskers show the 25th and 75th interquartile range and minimum–maximum, respectively; the median is the central line in each box. * indicates 0.001 < *p* ≤ 0.05.

Compared to HCs, the only circulating monocyte subset markedly increased in the whole patient group was the CD14^high^CD16^+^ subpopulation (*p* = 0.020) (Figure 4b–d, Table 3). Considering the clinical severity (CME and nCME subgroups) (Figure 5, Table 3), our analysis revealed that this increment was of statistical significance only with regard to CME patients (*p* = 0.05) (Figure 5c, Table 3), whereas in the nCME group, the intermediate CD14^high^CD16^+^ monocyte subset did not statistically differ in comparison to HCs (Figure 5c, Table 3). Also, among the patient cohorts, we did not observe any significant differences in the proportion of intermediate CD14^high^CD16^+^ monocytes (CME vs. nCME) (Figure 5c, Table 3). In turn, the classical CD14^high^CD16^−^ and non-classical CD14^high^CD16^+^ monocytes in PP were found to be statistically comparable to those obtained from HCs, irrespective of the clinical course (Figure 5a,b, Table 3).

Considering the associations among monocytes, negative relationships of classical CD14^high^CD16^−^ monocytes with non-classical CD14^high^CD16^+^ monocytes and intermediate CD14^high^CD16^+^ monocytes in PP patients were found (r = −0.99, *p* < 0.001 and r = −0.58, *p* = 0.027, respectively), whereas in the HCs, the association was statistically significant only with regard to the non-classical subset (r = −0.98, *p* < 0.001). Furthermore, the patient intermediate CD14^high^CD16^+^ subset was also correlated with non-classical CD14^high^CD16^+^ monocytes (r = 0.55, *p* = 0.040).

In turn, analysis of the associations between monocytic cells and CD4+ and CD8+ effectors of the adaptive immune responses revealed that the healthy Th1 subset correlated positively with non-classical monocytes and negatively with the classical subset (r = 0.71, *p* = 0.006 and r = −0.75, *p* = 0.003, respectively; Figure 3b), which is in contrast to the PP patient group, where the only significant association of Th1 cells was a positive one with intermediate CD14^high^CD16^+^ monocytes (r = 0.58, *p* = 0.028) (Figure 3a). There was no relationship of any monocyte subset with IL 17-secreting lymphocytes in all individuals studied (Figure 3a,b).

When considering the relationships with regulatory cell subsets, we observed that both the intermediate CD14^high^CD16^+^ and non-classical CD16^high^CD14^+^ monocytes from patients inversely correlated only with the Breg subset (r = −0.68, *p* = 0.007 and r = −0.70, *p* = 0.004, respectively) (Figure 3a). In contrast, the patient classical CD14^high^CD16^−^ subset was positively associated with Breg and CD16^−^IDO^+^ MDSC (r = 0.74, *p* = 0.002 and r = 0.54, *p* = 0.044, respectively), whereas no association was found with regard to Th1, Th17, Treg, Tsup, or the CD16^+^IDO^+^ subset (Figure 3a). In the HCs, no corresponding relationships of monocytes with regulatory cell subsets were observed (Figure 3b).

## 4. Discussion

The available research based on mouse ocular inflammatory models shows different patterns of inflammatory and resident myeloid cell populations [44]. Unfortunately, the authors do not confront these outcomes with PB findings. Although monocyte subpopulations in patients suffering from uveitis have previously been addressed, the authors do not provide the exact percentage of PP patients [40,45]. In this regard, our work adds valuable information to existing knowledge on circulating monocyte subpopulations in adult patients with PP, as it includes a homogeneous group of patients. Herein, we originally identified a specific profile in different circulating myeloid cell populations including peripheral expansion of CD16^+^ myeloid cells (both intermediate CD14^high^CD16^+^ monocytes and IDO-expressing CD16^+^ MDSC). Other monocyte subsets, such as classical CD14^high^CD16^−^ and non-classical CD14^+^CD16^high^, as well as CD16^−^IDO^+^ MDSC, were found unchanged regarding their percentages in the PB. Remarkably, despite a lack of statistically significant changes in the frequency of CD16^−^ myeloid cells, a shift toward a decrease (however non-significant) in the CD16^−^ fraction, corresponding to a simultaneous significant increase in CD16^+^ myeloid cells, primarily in the CME patient subgroup, has been observed. At this stage, we cannot exclude that the relatively small patient cohorts enrolled in our study could compromise the level of statistical significance obtained. In line with the present results, an increase in intermediate CD14^high^CD16^+^ cells has been found in the PB and/or inflamed tissues during chronic inflammation, including non-infectious uveitis [16,38,39,40,41,42,43,46,47,48,49,50].

Previous research has demonstrated several reasons for circulating intermediate CD14highCD16+ monocyte enrichment in chronic inflammation, including a transitory stage from classical to non-classical monocytes or maturation of CD16- to CD16^+^ cells [38,39,40,41,42,43,50]. In our study, the conversion of CD16- to CD16^+^ cells appears to be independent of the disease, as we observed an inverse correlation of the classical CD14^high^CD16- subset with CD16^+^ monocytes in all individuals studied. It was noticeable, however, that in HCs, classical monocyte negative association was assigned to the non-classical subset, whereas in patients, classical monocytes were also correlated with intermediate CD14^high^CD16+ monocytes. This might suggest that disease-dependent factors could enhance monocytes’ ability to acquire an intermediate level of CD16 expression. CD16 expression has previously been shown to be up-regulated on monocytes in response to affected levels of activated platelets, IFN-γ, IL-10, and TGF-β found in several inflammatory disorders and non-infectious uveitis [10,49,51,52,53]. Also, we cannot exclude that enrichment in the circulating intermediate CD14^high^CD16^+^ cell compartment only in patients with CME may reflect a strength of local inflammation and CD16- monocyte sequestration in the eye tissues. This process could be driven by the highest expression of receptor for a CCL2 chemokine (CCR2) found on the classical CD14^high^CD16^−^ monocytes [45,54]. Indeed, we noted no significant differences in the proportion of those subsets in the peripheral compartment between nCME patients and HCs. On the other hand, elevated levels of TNF-α found in sera from PP patients may also contribute to intermediate CD14^high^CD16^+^ monocyte peripheral accumulation by creating a microenvironment supporting their activation and differentiation [55]. Recent studies demonstrate that the intermediate CD14^high^CD16^+^ subset is extremely susceptible to TNF-α and, to a lesser extent, to chemokines, which is dependent on the corresponding level of TNF-α and chemokine receptors’ expression [54,56]. Given that the intermediate CD14^high^CD16^+^ subset correlates with inflammation markers and secretes increased amounts of TNF-α in chronic inflammatory diseases, it has been stated that this monocyte subset exerts a pro-inflammatory function [19,57]. Numerous other reports confirm the pro-inflammatory characteristics of CD14^high^CD16^+^ monocytes obtained from patients with chronic inflammatory diseases [16,19,54,56,57]. Although we did not find correlation of any monocyte subset with disease activity, it was noteworthy that the highest frequency of circulating intermediate CD14^high^CD16^+^ cells was found only in patients with CME. Also, our findings of their positive correlation with inflammatory Th1 cells remains in line with this notion, thus implying intermediate monocytes have a role in the propagation of the T effector cell response toward IFN-γ and TNF-α secreting cells. From a clinical point of view, the intermediate monocytes (regulated predominantly by TNF-α) can be a bridge to our understanding of why anti-TNF treatment has a high rate of success in inducting and maintaining the remission of ocular inflammation and other chronic inflammatory disorders [15,58,59].

IDO+ is an increasing MDSC subpopulation and accompanied by the lower accumulation of IDO within CD16^−^IDO^+^ MDSCs fraction in patients with CME only. However, given that we found no statistical differences in the size of the CD14^+^CD16-IDO^+^ and CD14^+^CD16^+^IDO^+^ populations in PP patients, and that the CD14^+^CD16- population is the predominant myeloid cell compartment in all participants, as it constitutes about 70% of circulating monocytes, we may conclude with caution that the absolute number of CD14^+^CD16^−^IDO^+^ cells in PP patients is higher compared to CD14^+^CD16^+^IDO^+^ cells. In consequence, the level of IDO in CD14^+^CD16^−^IDO+ cells seems to play a superior role in shaping the functional significance of the whole IDO+ MDSC population. Therefore, it seems reasonable to assume that the lower IDO level in the CD14^+^CD16^−^IDO^+^ we observed in the CME patient group might be responsible for the potential deficit in the regulatory function of the MDSC population in these patients, thus contributing to a more severe clinical course of PP. Since IDO+ MDSCs have been reported as essential for Treg induction and preventing the initiation of autoimmune disorders [26,35,60], decreased IDO levels in MDSCs from CME patients could explain, at least in part, regulatory T and B cell down-regulation in the same patient cohort. Although we did not perform functional assays of MDSCs, which is a weakness of our study, the above suggestion seems consistent with previous observations that the exacerbation of chronic inflammatory/autoimmune diseases is associated with inappropriate IDO activity [61,62,63,64,65]. Similar conclusions were also drawn from functional studies of MDSCs in the EAU [66]. In this experimental model of uveitis, it has been shown that suppression of EAU induced by accumulation of IDO in DCs could be reversed by 1-methyl tryptophan (1-MT), a pharmacological IDO inhibitor [66]. The above studies confirmed that amelioration of EAU is caused by an IDO-dependent mechanism. The reason for the compromised IDO level observed in the present study may be a deficiency in the Th1 cells producing IFN-γ detected in our patient cohort, since this cytokine was shown as a factor responsible for the accumulation of IDO in cells suppressing antigen-specific CD4+ T cells in EAU [66]. Furthermore, the associations of PP duration with the levels of both Th1 cells and CD14^+^CD16^+^IDO^+^ cells we identified seem to strengthen the above suggestion on the IFN-γ-mediated IDO expression in MDSCs in PP.

In contrast, the role of CD14^+^CD16^+^IDO^+^ cells in the functional activity of MDSCs appears less significant due to their negligible number in circulation. Nevertheless, it is worth emphasizing that only in the CME group was their percentage found to be significantly increased and exceeding the corresponding fraction of healthy MDSCs. This CD14^+^CD16^+^IDO^+^ increase in the CME group may suggest a counter-regulatory response of myeloid cells to the greater systemic immune activation and may merely reflect the IDO^+^ MDSCs’ attempt to compensate for the deficiency in the Treg population observed in CME patients. This corresponds to the demonstration that the IDO pathway provides a regulatory bridge connecting two opposite T cell subpopulations, namely regulatory T cells and effector Th17 cells [28]. In line with this, counter-regulatory response of CD16^+^IDO^+^ MDSCs increases with time, as we noted that the duration of PP correlated with CD16^+^IDO^+^ MDSC up-regulation and Th17 decrease. Furthermore, former data from uveitis and rheumatoid arthritis patients consistently showed an association of IDO pathway measured by increased kynurenine/tryptophan ratio, with elevated levels of neopterin, a marker of immune cell activation [67,68]. Therefore, it has been proposed to consider IDO+ subset as an indicator of the strength of the immune response or Th17-related inflammation [69,70]. In this regard, the results of the present study are in agreement with recent data showing that a fraction of circulating monocyte-like MDSC and CD16+ monocytes reflects the immunological and clinical responsiveness of inflamed patients [46,70]. It should, however, be emphasized that our conclusions regarding alterations in potential regulatory roles of IDO^+^ MDSCs in PP patients with clinical complications remain speculative and require confirmation in future dedicated functional studies.

## 5. Conclusions

Our study is one of the first to evaluate subpopulations of circulating CD16^+^ myeloid cells (including monocytes and MDSCs) in PP patients and the biggest to include a homogeneous group of patients. We have demonstrated that patients with PP clinically complicated by the presence of CME have a pronounced systemic innate and adaptive immune dysregulation, which may be considered as an indicator of the strength of local inflammation. Whether the observed immunological alterations in the PB could predict the severity of ocular inflammation or are secondary changes require verification in long-term studies on larger groups of patients.

## Figures and Tables

**Table 1:** Pro-inflammatory and anti-inflammatory lymphocyte subpopulations in the PB of PP patients and HCs.

Cell Population (%)	Patients (overall) (O) n = 15	Patients with Macular Edema (CME) n = 9	Patients Without Macular Edema (nCME) n = 6	Healthy Controls (HCs) n = 17	*p*-Values
CD3^+^CD8^−^IFN-γ^+^ (Th1)	4.89 (2.80–6.68)	5.35 (2.88–6.68)	4.44 (2.34–5.68)	8.43 (7.58–10.00)	O vs. HCs: <0.001 CME vs. nCME: 0.999 CME vs. HCs: 0.020 nCME vs. HCs: 0.014
CCR4^−^CXCR3^+^IFN-γ^+^ (Th1)	4.10 (3.06–5.28)	4.94 (3.23–5.28)	3.74 (3.06–4.39)	8.77 (5.13–9.71)	O vs. HCs: 0.006 CME vs. nCME: 0.999 CME vs. HCs: 0.058 nCME vs. HCs: 0.099
CD3^+^CD8^−^IL-17^+^ (Th17)	0.70 (0.60–0.94)	0.73 (0.62–0.78)	0.69 (0.60–0.94)	0.32 (0.22–0.48)	O vs. HCs: <0.001 CME vs. nCME: 0.999 CME vs. HCs: <0.001 nCME vs. HCs: 0.009
CCR4^+^CCR6^+^IL-17^+^ (Th17)	1.44 (1.02–2.03)	1.39 (1.02–1.71)	1.80 (1.44–2.03)	0.36 (0.26–0.45)	O vs. HCs: <0.001 CME vs. nCME: 0.999 CME vs. HCs: <0.001 nCME vs. HCs: 0.001
CD4^+^CD25^+^CD127^−^ (Treg)	2.34 (1.33–4.31)	2.31 (1.33–2.56)	3.35 (1.95–4.34)	3.90 (3.16–5.33)	O vs. HCs: 0.004 CME vs. nCME: 0.981 CME vs. HCs: 0.008 nCME vs. HCs: 0.421
CD4^+^CD25^high^CD127^−^ (Treg)	0.30 (0.11–0.44)	0.20 (0.11–0.44)	0.35 (0.16–0.43)	0.66 (0.51–1.10)	O vs. HCs: <0.001 CME vs. nCME: 0.981 CME vs. HCs: 0.004 nCME vs. HCs: 0.020
CD8^+^CD28^−^FoxP3^+^ (Tsup)	0.65 (0.24–2.64)	0.54 (0.39–2.35)	0.94 (0.22–2.69)	3.17 (1.75–5.51)	O vs. HCs: 0.005 CME vs. nCME: 0.988 CME vs. HCs: 0.045 nCME vs. HCs: 0.109
CD19^+^CD24^high^CD38^high^ (Breg)	0.98 (0.26–2.37)	0.82 (0.52–1.86)	1.25 (0.26–2.99)	1.35 (0.60–2.98)	O vs. HCs: 0.363 CME vs. nCME: 0.999 CME vs. HCs: 0.989 nCME vs. HCs: 0.989

Data are presented as medians (interquartile range). The *p*-values were derived from Student’s t-test and Mann–Whitney U test or one-way ANOVA and non-parametric one-way Kruskal–Wallis ANOVA, followed by Scheffe’s test or Dunn’s test, respectively. Significant results and trends are shown in bold.

**Table 2:** IDO expression in the MDSC subsets (CD16+ and CD16-) in PP patients and HCs.

IDO Expression in MDSC Subsets	Patients (overall) (O) n = 15	Patients with Macular Edema (CME) n = 9	Patients Without Macular Edema (nCME) n = 6	Healthy Controls (HCs) n = 17	*p*-Values
CD14^+^CD16^+^IDO^+^ (%)	0.45 (0.21–0.64)	0.58 (0.20–0.76)	0.40 (0.21–0.50)	0.11 (0.10–0.18)	O vs. HCs: 0.011 CME vs. nCME: 0.999 CME vs. HCs: 0.037 nCME vs. HCs: 0.204
CD14^+^CD16^−^IDO^+^ (%)	0.21 (0. 01–0.60)	0.21 (0. 01–0.37)	0.31 (0.10–0.60)	0.35 (0.15–0.65)	O vs. HCs: 0.333 CME vs. nCME: 0.999 CME vs. HCs: 0.652 nCME vs. HCs: 0.997
***p*-Values**	0.345	0.398	0.345	**0.016**	
IDO in CD14^+^CD16^+^ cells (MFI)	69.57 (54.38–108.43)	54.46 (49.89–108.43)	89.00 (54.75–122.61)	104.06 (78.65–204.45)	O vs. HCs: 0.169 CME vs. nCME: 0.999 CME vs. HCs: 0.141 nCME vs. HCs: 0.999
IDO in CD14^+^CD16^−^cells (MFI)	40.65 (0.00–62.93)	17.78 (0.00–42.58)	64.35 (41.83–74.99)	71.71 (32.45–233.38)	O vs. HCs: 0.071 CME vs. nCME: 0.202 CME vs. HCs: 0.041 nCME vs. HCs: 0.999
***p*-Values**	**0.071**	**0.028**	0.363	0.388	

Data are presented as medians (interquartile range). The *p*-values were derived from Student’s t-test and Mann–Whitney U test or one-way ANOVA and non-parametric one-way Kruskal–Wallis ANOVA, followed by Scheffe’s test or Dunn’s test, respectively. Significant results and trends are shown in bold.

**Table 3:** Monocyte cell subpopulations in PP patients and HCs.

Monocyte Subpopulation (%)	Patients (overall) (O) n = 15	Patients with Macular Edema (CME) n = 9	Patients Without Macular Edema (nCME) n = 6	Healthy Controls (HCs) n = 17	*p*-Values
CD14^high^CD16^−^ (CL)	72.12 (45.14–83.88)	68.21 (45.14–83.88)	76.02 (57.06–81.54)	66.92 (56.98–81.74)	O vs. HCs: 0.984 CME vs. nCME: 0.844 CME vs. HCs: 0.984 nCME vs. HCs: 0.817
CD14^+^CD16^high^ (NCL)	21.25 (13.53–46.97)	23.20 (13.53–46.97)	19.29 (14.23–35.59)	29.44 (15.92–37.72)	O vs. HCs: 0.796 CME vs. nCME: 0.981 CME vs. HCs: 0.999 nCME vs. HCs: 0.973
CD14^high^CD16^+^ (INT)	5.56 (3.57–7.63)	6.90 (3.57–7.88)	4.68 (4.22–6.44)	2.70 (2.20–4.91)	O vs. HCs: 0.020 CME vs. nCME: 0.999 CME vs. HCs: 0.052 nCME vs. HCs: 0.711

Data are presented as medians (interquartile range). The *p*-values were derived from Student’s t-test and Mann–Whitney U test or one-way ANOVA and non-parametric one-way Kruskal–Wallis ANOVA, followed by Scheffe’s test or Dunn’s test, respectively. Significant results and trends are shown in bold.

## Data Availability

The datasets generated and/or analyzed during the current study are available from the corresponding author on reasonable request.

## Supplementary Materials

The following supporting information can be downloaded at: https://www.mdpi.com/article/10.3390/cells14201636/s1, Table S1: Summary of the ophthalmic examinations performed with grading scales and specifications of the equipment used; Table S2: Detailed description of pars planitis screening group; Table S3: Inclusion and exclusion criteria for study enrolment; Figure S1: Gating strategy for the evaluation of the monocytes and IDO-expressing MDSC subpopulations.

## Abbreviations

The following abbreviations are used in this manuscript:

PP	pars planitis
PB	peripheral blood
MDSC	myeloid-derived suppressor cell
CME	cystoid macular edema
nCME	no cystoid macular edema
HC	healthy control
Th	T helper
IDO	indoleamine 2,3-dioxygenase
Treg	T regulatory
EAU	experimental autoimmune uveitis
TNF	tumor necrosis factor
IFN	interferon
IL	interleukin
BRB	blood–retina barrier
Tsup	T suppressor
Breg	B regulatory
LPS	lipopolysaccharide
PBMC	peripheral blood mononuclear cell
mAbs	monoclonal antibodies
PMA	phorbol 12-myristate 23-acetate
Ion	ionomycin
BFA	brefeldin A
CCR	C-C chemokine receptor
CXCR	C-X-C chemokine receptor
PerCP	peridinin-chlorophyll-protein
PE	phycoerythrin
FITC	fluorescein
FOXP3	Forkhead Box P3
BD	Becton Dickinson
M-MDSC	monocytic-like myeloid-derived suppressor cell
IQ	interquartile
TGF	tumor growth factor
1-MT	1-methyltryptophan
DC	dendritic cell
