# Supplementary material for: Circulating CD16-Positive Monocyte-like Myeloid-Derived Suppressor Cells and Intermediate Monocytes Associated with Clinical and Immunological Complications in Pars Planitis Patients
Source: Cells. 2025 Oct 21;14(20):1636. doi: 10.3390/cells14201636 (PMC12564362; doi:10.3390/cells14201636)
Supplement: Supplementary file 1 [file cells-14-01636-s001.zip › cells-3903939-supplementary.pdf]

Supplementary Materials

Table S1 Summary of the ophthalmic examinations performed with grading scales and specifications of the equipment used

- BCVA tested using ETDRS charts (different charts for each eye) from a distance of 4 m
- NV tested using Snellen charts from a distance of 25 cm
- Evaluation of the anterior segment of the eye (slit lamp examination) including assessment of the inflammation (anterior chamber cell count, anterior chamber flare count, vitreous haze scale according to SUN)
- IOP measurement performed with GAT
- Application of 1 drop of 10% Neosynephrin and 1% Tropicamid
- Assessment of lens opacity and vitreal inflammation
- Fundoscopy
- OCT examination of the macula and submacular choroid

BCVA – best-corrected visual acuity, IOP – intraocular pressure, GAT – Goldmann applanation tonometry

Table S2 Detailed description of pars planitis screening group

Test	Pars planitis screening group<br>N = 29
Borrelia burgdorferi	
IgG	
Positive/Negative	4/25
IgM and IgG	
Positive/Negative	1/28
Viral tests (for HSV-1 and HSV-2, VZV, HHV-7, CMV, EBV, and HIV)	
PCR<br>Positive/Negative	2/27
IgM<br>Positive/Negative	2/27
QuantiFERON/Skin reaction to tuberculin Mantoux test with tuberculin RT23)<br>Positive/Negative	3/26
Tuberculosis (vitreal samples)*	
Yes/No	1/28
Suspected systemic disease	
Yes/No	2/27
Withdrawal of written consent	
Yes/No	1/28

*Obtained after screening due to rapid bilateral exacerbation of symptoms.

Table S3 Inclusion and exclusion criteria for study enrolment

**Inclusion criteria**	**Exclusion criteria**
Age > 18 years old	Age <18 or > 70 years old
Written informed consent	No written consent or noncompliance with medical monitoring (study protocol)
Established diagnosis of pars planitis - according to Standarization of Uveitis Nomenclature Working Group (SUN) criteria:<br>1. Negative viral tests for HSV-1, HSV-2,VZV, HHV-7, CMV, Epstein-Barr virus, and HIV (commonly used tests)<br>2. Negative blood IgG and IgM levels for *Borrelia burgdorferi*, *Toxoplasma*, *Toxocara canis*, and *Bartonella pertussis*<br>3. Negative QuantiFERON and skin reaction to tuberculin (Mantoux test with tuberculin RT23)<br>4. Negative blood test for syphilis (Wassermann reaction and VDRL test)<br>5. No previous diagnosis of systemic autoimmune disease<br>6. No pathologic findings in an X-ray examination of the chest<br>Patient's observation for at least the time necessary to establish the diagnosis of pars planitis including diagnostic tests and other specialist consultation (rheumatological and neurological, for at least 4 weeks)	1. Presence of systemic diseases associated with intermediate uveitis: multiple sclerosis (defined by the McDonald criteria), evidence of sarcoidosis<br>2. Positive serology for syphilis, Lyme disease, *Toxocara canis*, and tuberculosis<br>3. Neoplastic disorder (diagnosed or suspected)<br>4. Pregnancy and breast feeding<br>5. Diabetes mellitus<br>6. Hypertension<br>7. Life-threatening diseases<br>8. Oral or local steroid treatment in the previous 4 weeks<br>9. Oral or local immunomodulatory treatment in the previous 6 months (such as methotrexate, azathioprine, mycophenolate mofetil, tacrolimus, cyclosporine, or cyclophosphamide)<br>10. Any previous biological treatment (e.g., adalimumab, infliximab, rituximab, and anakinra)

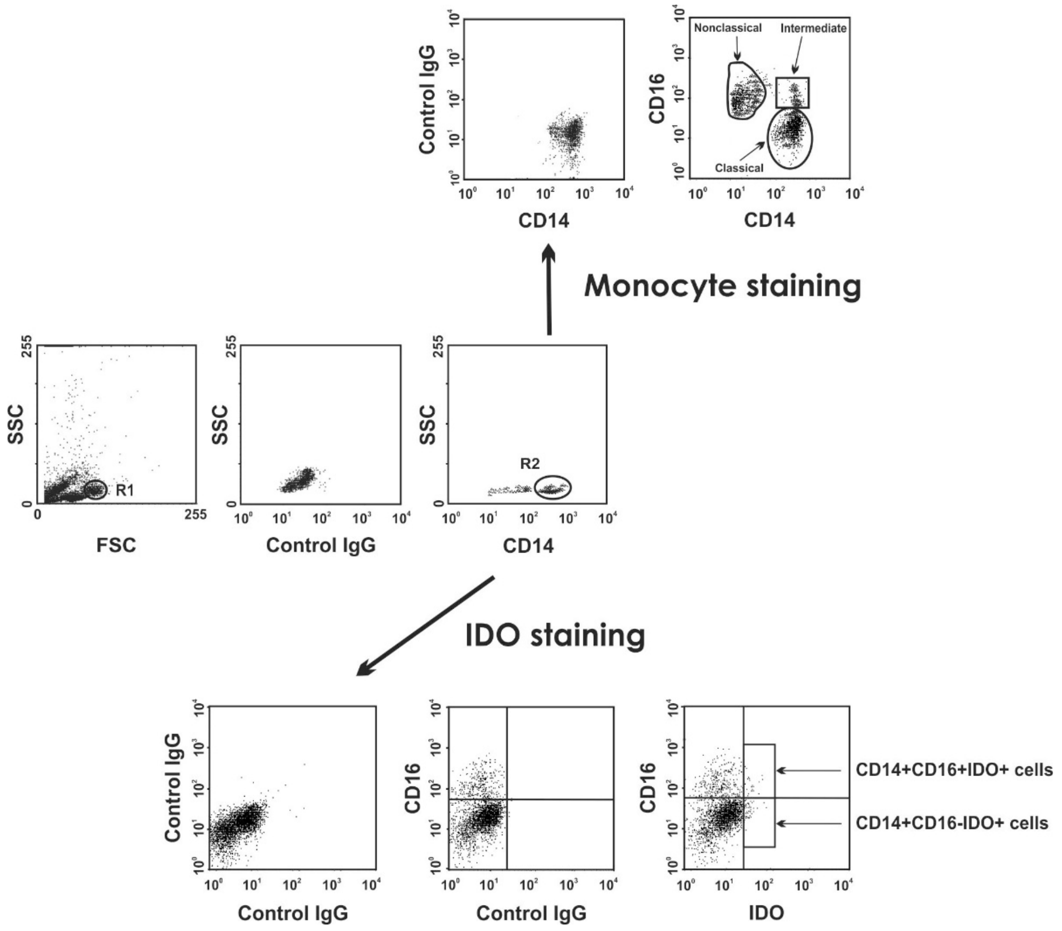

Figure S1. Gating strategy for the evaluation of the monocytes and IDO-expressing MDSC subpopulations. Representative plots demonstrating the analytic method for the identification of monocyte subpopulations (classical CD14highCD16-, intermediate CD14highCD16+, and non-classical CD14+CD16high) and IDO-expressing CD14+CD16+ and CD14+CD16- subsets. Monocytes were selected (R1 gate) based on their FSC/SSC properties. The R1 gate events were then analyzed for CD14 expression. CD14+ cells were gated (R2 gate). For analysis of monocytes subpopulations, gated CD14+ cells were analyzed for CD16+ expression using isotype control IgG for CD16 and CD16 MoAb. For analysis of IDO-expressing CD14+CD16+ and CD14+CD16- subsets, gated CD14+ cells were analyzed for CD16+ and IDO expression using isotype controls for CD16 and IDO, and CD16 and IDO MoAbs.
